# Mitochondrial RNA maturation

**DOI:** 10.1080/15476286.2024.2414157

**Published:** 2024-10-10

**Authors:** Zofia M. Chrzanowska-Lightowlers, Robert N. Lightowlers

**Affiliations:** Wellcome Centre for Mitochondrial Research, Biosciences Institute, Faculty of Medical Sciences, Newcastle University, Newcastle upon Tyne, UK

**Keywords:** Mitochondrial, messenger RNA, maturation, processing, modifications, translation

## Abstract

The vast majority of oxygen-utilizing eukaryotes need to express their own mitochondrial genome, mtDNA, to survive. In comparison to size of their nuclear genome, mtDNA is minimal, even in the most exceptional examples. Having evolved from bacteria in an endosymbiotic event, it might be expected that the process of mtDNA expression would be relatively simple. The aim of this short review is to illustrate just how wrong this assumption is. The production of functional mitochondrial RNA across species evolved in many directions. Organelles use a dizzying array of RNA processing, modifying, editing, splicing and maturation events that largely require the import of nuclear-encoded proteins from the cytosol. These processes are sometimes driven by the unusual behaviour of the mitochondrial genome from which the RNA is originally transcribed, but in many examples the complex processes that are essential for the production of functional RNA in the organelle, are fascinating and bewildering.

## Introduction

All cells require energy to complete their essential functions. In Eukarya, this energy is provided in the form of ATP, which can be generated by glycolysis or more efficiently synthesized by the process of oxidative phosphorylation (OXPHOS). The latter takes place in the mitochondrial network, a subcellular compartment that in addition to many other functions transduces approximately 90% of the cells energy, hence being referred to as the power house of the cell [1]. Surprisingly, although mitochondria share a single evolutionary origin [2] and the vast majority of eukarya harbour their own mitochondrial genome (mtDNA), there is virtually no single paradigm governing the details of mitochondrial gene expression that is universally conserved. Indeed, in almost all aspects of gene expression there is an inexplicable diversity of mechanisms to elicit the same functions, underlining the idiosyncratic nature of these organelles. The theme of this review is the maturation of mitochondrial (mt-) RNA. However, the huge *trans*-species variation in mitochondrial gene expression starts with the mtDNA itself. These organelles retain a vestigial genome that complements the content of the nuclear DNA. Mitochondrial DNA can vary in size, shape, form and mode of replication or ability to recombine, depending on the organism [3]. Moreover, the genetic composition of these different mitochondrial genomes varies in the presence or absence of untranslated regions or introns. Generally, however, a core of genes, in most animals at least, is similar even if the arrangement differs. These include genes encoding mt-rRNAs components of the mitoribosomes, mt-mRNAs encoding polypeptides most of which are part of the multi-complex OXPHOS machinery, and mt-tRNAs required for the intra-mitochondrial protein synthesis encoded by the mt-mRNAs. Which of the OXPHOS genes are encoded may vary; for many animal species this is 13, seven for complex I, one for complex III, three for complex IV and two for complex V (reviewed in [4]). Indeed, mtDNA from most eukarya encodes a similarly small number of proteins. The unicellular protist *Reclinomonas americana* is something of an outlier, with the mtDNA encoding a diverse repertoire of almost 100 proteins [5]. This panoply includes 31 structural RNAs and 65 proteins many of which are not usually encoded by mtDNA, such as the ABC or twin arginine transporters [6].

Another example of the complexity is in the number and source of tRNAs used for mitochondrial protein synthesis. There is also considerable heterogeneity in which tRNA genes have been retained in the mitogenome. Human mtDNA expresses only 22 tRNAs but these represent the full complement necessary for intra-mitochondrial protein synthesis. Such a small number is sufficient due to these unusually malleable mitochondrial tRNAs being able to tolerate some mismatch (‘wobble’ hypothesis) within the codon:anticodon base pairing [7]. A similarly small pool of tRNAs is used for synthesizing mitochondrial proteins in many different species. The budding yeast *S. cerevisiae* also encodes a complete set of 24 tRNAs for intramitochondrial protein synthesis but is reported to import a single tRNA from the cytosol, the lysine isoacceptor tRNA^Lys(CUU).^ This is postulated to be important for mitochondrial protein synthesis under certain stress conditions [8]. In contrast, no mitogenome from angiosperm species encodes a tRNA for every amino acid [9]. Typically, flowering plants encode 11–13 mt-tRNA genes which need to complement a variable number of nuclear encoded and imported tRNAs to ensure accurate intramitochondrial protein synthesis [10]. Trypanosomes represent a more extreme example in that there are no tRNAs encoded in their mitochondrial genome. The full complement of tRNAs are nuclear encoded and need to be imported into chloroplast or mitochondria to promote translation of the organellar genome [11].

Besides encoding a hydrophobic set of OXPHOS polypeptides, yeast mtDNA express one mitoribosomal protein from their mtDNA repertoire (Var1). A further approximately 1200+ proteins are needed to generate a functional mitochondrial network in addition to the mtDNA encoded proteins. Thus, the vast majority are nuclear encoded and are synthesized on cytosolic ribosomes prior to post- or co-translational import into the mitochondrial network [12]. Amongst these are the proteins required to transcribe the mt-genome, as well as those that facilitate the production of functional mt-RNA molecules.

## Transcription of mtDNA

Transcription is effected by a mitochondrial RNA polymerase. In most organisms, including yeasts, plants, trypanosomes and mammals, this is a single subunit phage-like enzyme [13,14]. It acts with assistance from factors that help to open and unwind the mtDNA and facilitate the interaction of the polymerase with the template [15,16]. Many aspects of mitochondrial transcription differ across species. The number and position of the promoters within the mitochondrial genome varies, with one or multiple promoters per strand. In some insect mtDNA, promoters have not been fully identified and from the analysis of extensive deep sequencing of multiple invertebrates it is likely that several transcription initiation and termination sites exist in most invertebrate mitochondrial genomes [17]. Only very recently have the transcription initiation and termination sites in mtDNA from a number of *Drosophila* species been mapped with any certainty [17]. For trypanosomes, the situation is more complicated as the genome is spread between multiple maxicircles and minicircles, the sizes and numbers of which are constant within, but vary markedly between, species [11]. Polycistronic RNAs that encompass almost the entire mt-genome are found in a number of eukaryotes. Initially, this was also thought to be the case in trypanosomes [18]. This assumption has been challenged, however, as recently developed techniques have shown trypanosomal mtDNA transcription to proceed on the maxicircles via independent synthesis of individual immature mRNAs and rRNAs. Similarly, the small guide RNAs (which serve as templates for RNA editing) encoded on the minicircles are transcribed from dedicated promoters [19].

**Figure 1.**
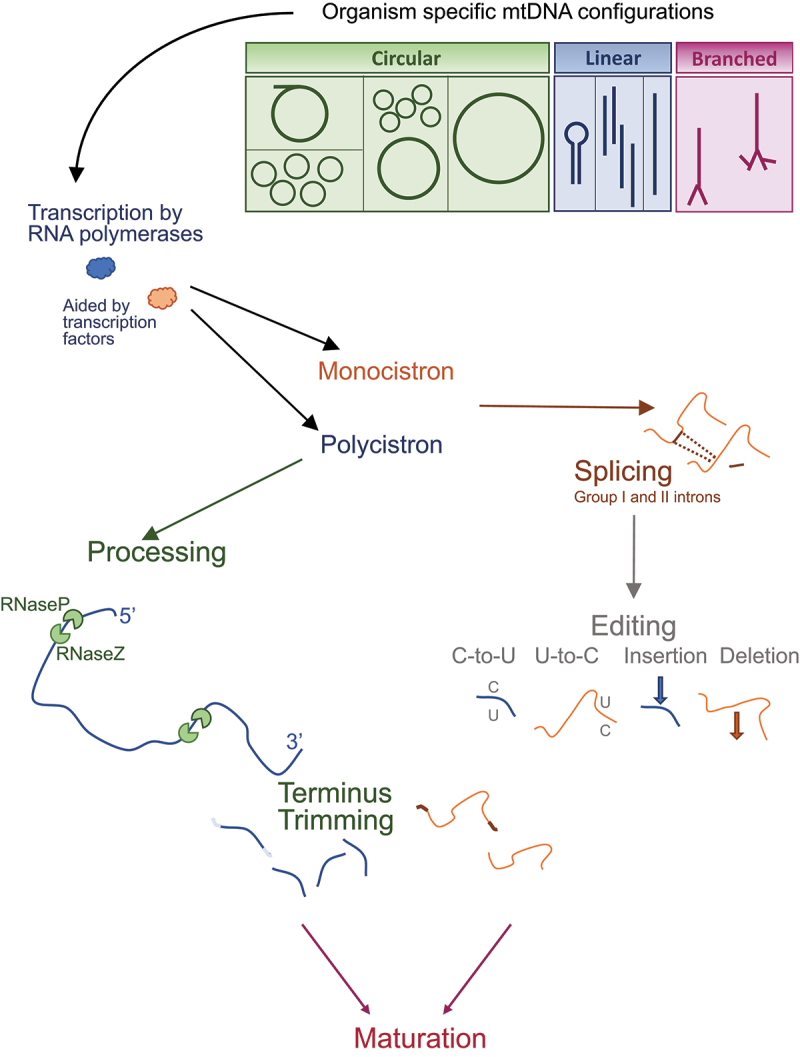
Mitochondrial DNA (mtDNA) can vary in form and different structural variants are presented in this cartoon. These can be found as linear, branched, as various combinations of circular forms, or closed circular molecules as found in many mammalian species, depending on the organism almost every stage of preparing transcripts for use in translation varies. **transcription** can be from a promotor that leads to a transcript that encodes multiple genes (polycistron) or from promotors that are each responsible for a single gene product (monocistron). The co-transcriptional incorporation of an NAD+ cap occurs on a subset of RNAs in a subset of organisms. The former will need to be processed into the individual rRNA, tRNA or mRNA species. For transcripts that contain introns these are removed by **splicing**. Generation of complete open reading frames may require **editing** that has different forms. Individual species may then require the 5’ and 3’ ends to be **trimmed.**

The composition of transcription units continues the divergent nature of gene expression, as immature RNA species may encode either a single gene (monocistronic RNA unit) or several genes in one transcript (polycistronic) (Figure 1). Subsequently, these RNA species may or may not require processing or splicing using non- or canonical splice sites, or *trans*-spliced transcripts (see below). The variation between organisms continues within the mt-mRNA species as some may contain noncanonical translation starts, have altered codon usage, stop-codon readthroughs, multiphasic coding exons or use ribosomal frameshifting.

## Mitochondrial RNA processing

In the context of this review, processing will be defined as ribonucleotide excision and trimming from transcription units. Polycistronic transcripts need to be separated into their multiple components before they can be fully matured in various ways and be available for use in protein synthesis. In many species, the gene arrangement is such that the open reading frames and ribosomal RNAs are interspersed with tRNAs. The method of release of the tRNAs from the larger transcription unit has been described via a punctuation model. Cleavage occurs in a specific order, generating sequentially smaller transcription units [20]. In mammals, the mt-tRNAs are believed to fold into a cloverleaf structure that is recognized to trigger processing. This is performed by two endonucleases; RNase P, a heterotrimeric complex devoid of RNA that cleaves 5’ of the mt-tRNA. The second enzyme is an RNase Z (ELAC2) and performs the downstream cleavage at the 3’ end of the mt-tRNA ([20,21] and reviewed in [22]) (Figure 1). The few non-canonical mt-mRNAs that are not flanked by mt-tRNAs are processed by a different mechanism that is likely to involve members of the **F**as-**a**ctivated **s**erine/**t**hreonine **k**inase (FASTK) family (reviewed in [22]) and a 3’ phosphatase, Angel 2 [23]. In humans, the enzyme YbeY appears to facilitate the accurate processing of both the 5′ and 3′ ends of at least the tRNA^Ser(AGY)^ [24]. This additional role differs from that in bacteria [25,26] or plant chloroplasts where the activity of the orthologue is restricted to trimming rRNA species [27].

Mitochondria from across the plant kingdom largely also generate long polycistronic RNA precursor transcripts that need to be processed into smaller units [28]. In a manner analogous to that used in human mitochondria, the endonucleolytic excision of tRNAs in *A. thaliana* is performed by a single subunit RNase P (PRORP1), which also matures the RNA 5’ terminus by removal of any RNA leader sequence [29]. The 3’ terminus of the tRNA is matured by TRZ2, one of four RNase Z enzymes present in the plant cell. Although these are the main enzymes responsible, their activity is modulated by a number of other factors (reviewed in [30]).

Unlike their mammalian counterparts, *Drosophila* mt-RNA is transcribed from multiple promoters. Transcription still forms polycistronic units, which undergo successive cleavage of the 5’and 3’ ends of mt-tRNAs by RNase P and RNase Z complexes [31]. Similarly, mtDNA both from budding (*S. cerevisiae*) and fission (*S. pombe*) yeast, is transcribed from multiple promoters. Primary transcripts comprise polycistronic units, often with long noncoding regions [32,33]. The precursor RNAs still need to be cleaved into smaller units. Initial steps in the production of functional tRNAs also require 5′- and 3′-processing of these precursor tRNAs (pre-tRNAs). As is the prevalent model, this is performed by the two endonucleases, RNase P and RNase Z. In this yeast model, they may constitute part of a much larger complex [34]. The RNase P complex has been reported to require 4 ancillary factors to ensure efficient excision of mt-tRNAs [35]. It comprises a protein encoded by the nuclear *RPM2* gene and the mtDNA-encoded 9S RNA component [36]. Composition can vary across different yeast species and not all complexes contain the RNA component (*C. albicans* [37]). In *S. cerevisiae*, after separation from the larger transcription unit, pre-mRNAs require a further cleavage that occurs downstream of a dodecamer motif [38]. The responsible enzyme is yet to be identified. The cleaved 3’ terminus is then protected from degradation by the RNA-binding protein Rmd9p [39]. This pattern does not appear to be conserved across the yeast families as no dodecamer or equivalent motif has been identified in *C. albicans* or other budding yeast distantly related to *S. cerevisiae*, although curiously *C. albicans* has retained an orthologue of Rmd9.

In certain cases, both mono- or polycistronic RNAs may need some form of additional trimming of the 5’ or 3’ termini (Figure 1). For example, in addition to the processing of yeast polycistronic RNAs described above, some transcripts such as *COB* require further trimming of 5’ termini by Cbp1p and Cbt1p. These proteins act in concert to generate the mature terminus and protect it from the exonuclease Pet127p. This complex may involve interaction with RNase P (reviewed in [40]).

Mitochondrial transcripts from vascular plants can originate from one of multiple promoters that initiate in a way that generates a mature 5’ end with no need for further processing [41]. This is not the case in *A. thaliana*; however, where for most mtDNA transcripts the 5′ termini are trimmed by the combined action of pentatricopeptide repeat-containing (PPR) RNA-processing factors and endonucleases (reviewed in [30]). For trypanosome transcripts, this further processing depends on whether the transcribed RNA species derive from the maxicircles or the minicircles, but both are generated initially as 3′-extended precursors which undergo exonucleolytic trimming. Maxicircles encode the mt-rRNAs and -mRNAs that are commonly encoded by mtDNA. The 5′ termini of the derived RNA species are defined via transcription, but similar to the situation with the minicircle-encoded gRNAs, their 3’ termini carry extensions. Both termini are subsequently matured as described below. The minicircles, in contrast, encode gRNAs that direct the subsequent U-insertion/deletion mRNA editing (also below). The 5’ end of these transcripts retain the triphosphate from transcription initiation, but the processing of the 3’ terminus involves uridylation followed by 3’-5’ exonucleolytic processing to define the 3’ end [42].

## Mitochondrial RNA editing and pentatricopeptide repeat containing (PPR) proteins

RNA editing appears to have evolved multiple times, potentially recruiting existing enzymes that originally had unrelated activities. Such mt-RNA editing has been identified in mitochondria from diverse organisms of various complexities, including dinoflagellates, kinetoplastids, fungi, land plants and animals (reviewed in [43]). It may serve a variety of purposes such as to (i) change ACG start codons to AUG, thus altering the efficiency of translation and thereby fine tuning the proteome, (ii) alter the structure of an RNA species, affecting its recognition, (iii) contribute to splicing efficiency, (iv) complete a reading frame for expression of a polypeptide, or (v) signal the readiness of an mRNA for translation. In trypanosomes, most of the protein-coding transcripts must undergo extensive RNA editing (Figure 1). These post-transcriptional events can take the form of insertions or deletions of nucleotides, or result in base substitutions or modifications. This process was first identified over 35 years ago and presented an example of how editing an mRNA completed an interrupted open reading frame. Original interrogation of the sequences detected a discrepancy between the DNA and RNA sequences encoding cytochrome *c* oxidase subunit 2 of *T. brucei* [44]. This highly conserved mtDNA-encoded gene and its transcript appeared to misalign, until it was recognized that there was an insertion of 4 uncoded UMPs into the transcript, allowing the production of a full-length protein [44]. Thus, in the idiosyncratic way of mitochondria, *T. brucei* employs a different means to achieve the same end of a full length, in frame, translation competent mRNA. The underlying molecular mechanisms that were responsible in kinetoplastids began to be characterized five years later when Simpson and colleagues were able to show that short RNA species encoded within the intergenic regions of the maxicircles were responsible for ‘guiding’ the editing machinery to areas of sequence complementarity in the primary transcripts [45]. RNA editing of mitochondrial transcripts in protist and also plant is extensive. Hundreds to thousands of residues within trypanosome mt-mRNAs are edited through multistep gRNA-directed U insertions/deletions. These events can not only correct frameshifts in kinetoplast mRNAs but have the capacity to introduce translation punctuation signals [46]. Novel insertions continue to be identified [47].

Mechanisms of RNA editing can differ considerably between animals and plants. Interestingly, editing seems to be restricted to land plants and has not been observed in green algae [48]. As mentioned above, trypanosomes can insert or delete hundreds of uridines within sequences, whereas land plant mitochondria tend to edit via site-specific C to U deamination [49] and can convert thousands of cytidines into uridines [50,51]. Such editing occurs mainly in exon sequences to restore the canonical protein coding capacity of immature mitochondrial RNAs [52] but also in intronic sequences, often changing RNA conformation to facilitate splicing [53]. The C to U conversion is in part mediated by a class of RNA-binding PPR proteins that form interactions to generate a higher order ‘editosome’ (reviewed in [49,54]). This group of proteins, identified more than 30 years ago, harbour tandem arrays of between 2 and 30 repeating structural units that form an interaction with each target sequence, dictating specific single-stranded RNA-binding and recognition properties [55–57]. These proteins are represented in mitochondria from many different organisms but have expanded most prolifically in plants. Many individual species can harbour hundreds of unique PPR proteins. *Selaginella* exhibits over 2000 editing sites perhaps explaining the need for a remarkable 1500+ members of the PPR family, targeted either to mitochondria or chloroplasts [58–61]. Specific editing sites have also been identified in both exonic and non-coding intronic plant mitochondrial transcripts that need to be edited before correct splicing can be triggered. Although introduced in this section on RNA editing, PPR proteins are involved in several other stages of post-transcriptional RNA handling, such as processing, maturation, stability, and regulation of translation but also intron splicing [57].

PPR proteins are also found in human mitochondria but these are few. They are involved in transcription, 5’ cleavage and excision of mt-tRNAs, mt-mRNA stability, or translation (reviewed in [62]). They are not involved in splicing, as the compact nature of the mammalian genome means that introns are lacking, negating the need for splicing machinery. As with mammals, yeast also encode relatively few PPR proteins (10 in *S. pombe* and 15 in *S. cerevisiae*) all of which are targeted to mitochondria [63]. The functions in *S. pombe* appear more to be restricted to stability or activation/repression of translation [64]. This is also true of *S. cerevisiae* where Aep2/3 stabilize and activate *ATP9* or *ATP6/8* mRNAs respectively [40]. Additionally, the PPR protein Pet309 stabilizes intron-containing alleles of COX1 mRNA, whilst also being indirectly involved in splicing [63].

## Mitochondrial RNA splicing

RNA splicing occurs in all domains of life to remove intronic RNA sequences from within immature transcripts. Several different mechanisms have been identified, but some RNAs, including mitochondrial RNAs, remove group I or group II introns via a self-catalytic process. The two groups differ in their exact mechanisms of autocatalysis and splicing, which are detailed in [65]. These autocatalytic molecules constitute mobile genetic elements, often encoding reverse transcriptases or proteins that facilitate the splicing event. Splicing mechanisms to remove group I and II intronic sequences from yeast mitochondrial RNA and the importance of their intron-encoded maturases were described over 40 years ago [66]. Since then, a number of nuclear encoded splicing factors and their mitochondrial substrates have been characterized (reviewed in [40]). In some cases, an RNA chaperone such as a DEAD-Box helicase is necessary to ensure the correct RNA structure for efficient splicing [67]. In the case of the *cob* and *cox1a* genes, a key component is involved that surprisingly turned out to be the mitochondrial leucyl-tRNA synthetase LARS2 [68]. *Neurospora crassa* has also recruited an aminoacyl-tRNA synthetase to promote splicing, but it is the mitochondrial tyrosyl-tRNA synthetase YARS2 that is involved in slicing group I introns [69]. Group II introns are less frequent than group I in yeast mitochondria but do occur [70].

Group I and group II introns are present in land plants. In contrast to most other mitochondrial systems, however, the latter are more prevalent in plant mitochondrial genomes [71]. Intron content is highly variable in immature transcripts ranging from 4 to over 40 [72,73] but content appears relatively stable within each lineage [74]. The removal of introns from immature mt-mRNAs normally requires either *cis*- or *trans*-splicing (joining RNA sequences from two different primary transcripts), with *trans*-splicing evolving numerous times in vascular plant lineages [74]. An example of the latter is found in *Arabidopsis* where the 5 exons of the *nad1* transcript are interspersed with other genes across more than 175kb of mtDNA. *Trans*-splicing is carried out by the nuclear encoded PPR protein OTP43 in order to generate the uninterrupted open reading frame [75]. *Selaginella* also utilizing a splicing peculiarity not common in other plant mtDNA, whereby the *nad*4 L gene is located in the same transcriptional orientation within a group II intron of the *nad*1 gene [51]. Here, the excision of the nad4L sequences from the nad1 transcript has the twofold benefit of maturation of both of the complex I RNAs [51].

The endonucleases and maturases responsible for mt-RNA splicing are often encoded within the group I and group II introns respectively. Although group II infers self-splicing, this process requires a plethora of additional RNA binding proteins helicases, maturases and PPR proteins to enhance this process and can play important regulatory functions. Indeed, recent work has suggested that in rice, the PPR protein SOP10 modulates both intron splicing and editing of various transcripts encoding components of complex I to enhance cold tolerance [76]. In angio- and gymnosperms, most mitogenomes contain a single conserved maturase orthologue, metR, located in an intron of NADH dehydrogenase 1 that is involved in the splicing of introns from various pre-mRNAs [77]. Previous work has shown that the maturation of mitochondrial pre-mRNA in *Arabidopsis* involves both a maturase, nMAT2, and an RNA helicase, PMH2, to splice a subset of group II introns but as part of a larger spliceosomal complex [78]. The additional factors involved may be specific to a single intron or involved in the splicing of multiple introns (reviewed in [30]).

There is a surprising evolutionary divergence in the mitochondrial RNA processing of three related Euglenozoan species. Unusually, as well as relying on extensive RNA editing, Diplonemid mitochondria engage in *trans*-splicing of RNA, necessary as short RNA species are transcribed from many small mtDNA chromosomes. The second group, Kinetoplastids have focused solely on RNA editing. In striking contrast, mitochondria from a third group of Euglenozoa show no need at all for either editing or *trans*-splicing of RNA ([79] and reviewed in [80]).

## Maturation and modification steps

**Figure 2.**
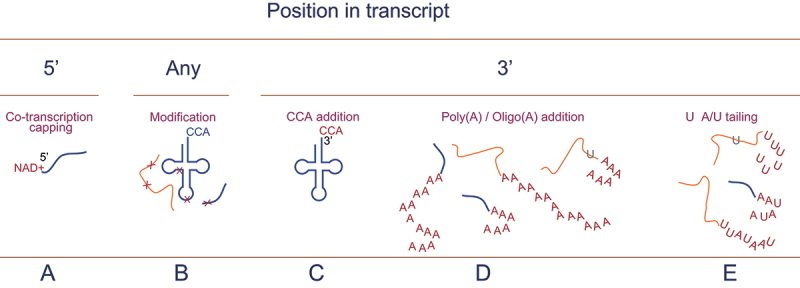
The final stages of transcript preparation prior to use is maturation. This can be by the addition onto the 5’ terminus as described in Figure 1 (A); co-transcriptional addition of an NAD+ cap) or decoration of the 3’ terminus with different nucleotide combinations depending on the type of RNA. Whilst tRNAs receive CCA tails (C), in mRNAs these can be oligo(a), poly(a) or poly(u/a) (D, E). Further **maturation** takes the form of different modifications dependent on the organism that are found rRNA, tRNA and mRNAs (B). Modifications depicted in burgundy; unspliced RNA in blue; spliced RNA in orange; edited nucleotides in grey.

Post-transcriptionally, all three functional groups of mt-RNA require base modification as part of their maturation process (Figure 2). To date over 160 post-transcriptional RNA modifications that affect either nuclear or mitochondrial RNA species have been described [81,82]. Since this is so wide an area to cover, this review will limit comments to the most frequent, characteristic or associated with pathogenicity. Following processing, maturation of immature mt-tRNAs from yeast and human involves modification at their 3’ termini by the addition of an untemplated CCA trimer. This essential process is facilitated by the [CCA]-tRNA nucleotidyltransferase, TRNT1. A single gene encodes the protein that is found both in the cytosol and the mitochondrial matrix [83,84]. Unsurprisingly, TRNT1 mutations are known to cause profound neonatal defects associated with mitochondrial disease [85]. Several studies over the years have indicated that a human mitochondrial protein, presumably mitochondrial poly(A) polymerase, can aberrantly oligoadenylate free 3’OH termini from various RNA sources [86–88]. Removal of these oligo(A) sequences from the 3’ termini of mt-tRNAs is essential for mitochondrial translation and is facilitated by PDE12 [89]. In the protist *T. brucei*, all tRNAs used for mitochondrial protein synthesis need to be imported. A single enzyme responsible for CCA addition to the *T. brucei* tRNA (TbCAE) [90] has been characterized and is found in all compartments of the cell. Intriguingly, all imported tRNAs are already aminoacylated, raising the prospect that the enzyme may have a second function in the mitochondria.

Human mitochondrial tRNAs have multiple modification sites which contribute to their stability and structural integrity. Recent mapping in human placenta and HeLa cells has characterized the complete panoply of human mt-tRNA modifications [91]. Across the 22 mt-tRNAs there are 137 positions that are modified by 34 different proteins responsible for 18 types of modification (comprehensively reviewed in [91]). Hypomodification at many of these sites has been associated with mitochondrial diseases [92–94]. Examples include loss of 5-taurinomethyluridine (τm^5^U) causing mitochondrial encephalopathies or MELAS (Mitochondrial myopathy, Encephalopathy, Lactic Acidosis and Stroke-like episodes) and fifteen pathogenic mt-tRNA mutations have been characterized that impair *N*^6^-threonylcarbamoyladenosine modifications and cause symptoms of MERRF syndrome (Myoclonic Epilepsy with Ragged Red Fibres) [92,95].

In addition, 5-formylcytidine, found only in mammalian mitochondria, is required to facilitate translational initiation from the non-canonical AUA start codons. The two methyltransferase complexes (TRMT6/61A and TRMT61B) are amongst those that bring about this modification in mt-tRNAs are also responsible for adding over twenty m1A modifications across mt-mRNAs [22,96,97]. This modification has been reported to impede translation by blocking the A:U base pairing with the cognate mt-tRNA [22]. Such stalling of the mitoribosomes has been demonstrated in *MT-ND5* [97] where the modification is added by TRMT10C [98]. Variation in the levels of this modification are associated with multiple disease phenotypes [99]. Another modification found in human mt-mRNAs is the conversion of specific uridines into pseudouridines effected by the pseudouridine synthetase complex including RPUSD3 and TRUB2 [100,101]. it is not yet clear what molecular function this modification confers.

Plants also utilize methylation modifications. N6 -methyladenosine (m^6^A) methylation has been found on angiosperm mitochondrial mRNAs [102,103] but the proteins responsible are yet to be identified (reviewed in [104] and [105]). Modifications at the 5’ termini of transcripts have also been reported. For example, transcripts from *Arabidopsis thaliana* can be capped at their 5’-termini, generating 5’ NAD^+^ RNAs [106]. This modification was found on nuclear and mitochondrially encoded transcripts and is thought to be incorporated as the initiating nucleotide rather than as a post-transcriptional modification. Splicing and polyadenylation of these modified species proceeds normally and association with ribosomes demonstrated that the modification did not impede translation of the encoded transcripts [106]. This modification has also been reported for a subset of mitochondrially encoded transcripts from *S. cerevisiae* where it too appears to be added co-transcriptionally [107]. The human mitochondrial RNA polymerase (RNAP) has been shown *in vitro* to be able to co-transcriptionally incorporate this modification and analysis of mitochondrial transcripts from HEK293T cells showed a subset of these to be NAD^+^ capped [108]. This potentially provides RNAP with a role of metabolic sensor and an additional mechanism for regulation of gene expression (reviewed in [109]).

Relatively little is known about mt-rRNA maturation in plants [78] but m^6^A methylation of 18S rRNA does occur in *Arabidopsis* [102], brought about by the rRNA dimethyladenosine methyltransferase DIM1B [110]. Mapping of human mitochondrial ribosomal RNAs has identified a modest ten modifications, although other low-level modifications cannot be ruled out [111]. Most are positioned in highly conserved sites that are critical for mitoribosome function (reviewed in [112]). All occur as nucleobase modifications: pseudouridylation, methylation or 2’-O-methylation. Unlike their mainly conserved nuclear-encoded counterparts, none are facilitated by sequence-dependent small nucleolar RNAs but require specific enzymatic activities. Indeed, the enzymes responsible for all ten modifications of mt-rRNA are now known with most of the enzymes responsible having bacterial homologues [113]. Surprisingly, depletion of the last of these proteins to be identified, TRMT2B, has no measurable effect on mitochondrial protein synthesis [114]. Although in general the modified bases show strong sequence conservation, the exact functions of these modifications are unknown [115]. Yeast mt-rRNA is even more modestly modified with only two 2’-O-methylated bases being introduced by the methyltransferases Mrm1p and Mrm2p [116] and 1 pseudouridine effected by PUS5 [101,117]. All are found in the large 21S mt-rRNA.

Modification of protist mt-rRNA or -mRNA modification has not been extensively studied, although it is clear that non-templated uridylation at the 3’ termini of both 9S and 12S mt-rRNA of *T. brucei* does occur [118]. Recently, searching for orthologues of mitochondrial pseudouridylate synthase genes in *T. brucei* revealed two contenders. The absence of one of these proteins, mt-LAF3, has been shown to cause depletion not only in both mt-rRNAs but also in mt-mRNAs. Extended studies revealed that the catalytic function of this PUS orthologue was not required, leading the authors to speculate that a paralogue, Tb927.3.2130, with similarity to the human active enzyme RPUSD4 may work in tandem with mt-LAF3 to protect and stabilize mt-RNA substrates [119]. Following initial processing, most trypanosome transcripts need some further steps of maturation or modification. The transcription-determined 5′ termini of trypanosome maxicircle-derived transcripts for example, are subsequently monophosphorylated by the pyrophosphohydrolase complex, ‘PPsome’ [19]. This is a tripartite complex comprising the MERS1 NUDIX enzyme, MERS2 the PPR RNA-binding subunit, and MERS3 polypeptide. The complex binds to specific sequences near mRNA 5′ termini. In contrast, most minicircle encoded guide RNAs lack PPsome-recognition sites and remain triphosphorylated [19]. Tethering of the 5’ and 3’ termini is facilitated by the cap binding of the PPsome interacting with the kinetoplast polyadenylation complex. The 3’-terminus is also trimmed but through an anti-sense non-coding RNA-mediated mechanism prior to adenylation [19] by the KPAP poly(A) polymerase.

## Polyadenylation

Remarkably, it is now fifty years since Hirsh and Penman were able to show that mitochondrial mRNA isolated from human HeLa cells contain non-templated 3’ polyadenylic acid extensions of roughly 50 nucleotides [120]. We now know that of the eleven mt-mRNA species, only *MT-ND6* (the sole mt-mRNA encoded by the L-strand) does not carry a poly(A) extension. While polyadenylation on mitochondrial transcripts has been suggested to be a two-step process, at least the major part of these extensions are synthesized by a mitochondrial poly(A) polymerase (mtPAP) although it is unlikely that mtPAP is sufficient to drive polyadenylation of mt-mRNA alone (see below) [121,122]. Intriguingly, analysis of steady state mt-mRNAs routinely reveals two populations for each species, oligoadenylated and fully polyadenylated populations. The relative levels of these two populations can vary between mt-mRNA species and between tissues [123]. Poly-, or oligoadenylated RNA is found in all domains of life. In mammals, polyadenylation of cytosolic mRNA generally increases the stability of the messages In bacteria, however, oligoadenylation of RNA is an essential step towards RNA degradation [124]. Critically, in the mitochondrion, orthologs of central players in either polyadenylation-mediated translation (poly(A) binding protein PABC paralogs; cap-binding proteins eIF-4e and eIF-4F), or decay (endoribonuclease RNase E) are absent.

What role, therefore, is played by poly- or oligoadenylation of RNA in the mitochondrial network? This apparently simple question has proved difficult to fully resolve. One function is clear. As mentioned above, mtRNAs are available for maturation subsequent to their endonucleolytic release from larger RNA precursors. It is necessary that at least oligoadenylation occurs at the 3’ termini of 7 of the 11 mammalian mt-mRNA species, as the 3’ cleavage does not leave a complete termination codon at the end of the open reading frame. In *Drosophila melanogaster*, 3’ polyadenylation appears to be a one, rather than two, step process and is necessary for the production of termination codons of incomplete orfs. Following the loss of mtPAP in *Drosophila*, mt-mRNA stability was unaffected. Translation of the nonadenylated species still occurred but was mildly aberrant [31]. Curiously, the maturation of mt-tRNA^Cys^ was affected, suggesting a role for polyadenylation in maturation/stability of specific mt-tRNAs [31]. Polyadenylation of mammalian mt-mRNAs, however, appears to have a more complex function. A generic RNA-binding protein the Leucine Rich PentatricoPeptide Repeat Containing protein (LRPPRC) has an essential function in stabilizing mt-mRNA and – rRNA [125]. On entering the mitochondrial network it associates with a second, smaller protein, the SRA stem-loop interacting RNA binding protein, SLIRP to form a complex that is mutually dependent for protein stability. On depletion of LRPPRC, mt-mRNA rapidly decays and polyadenylation is lost. This observation is consistent with *in vitro* reconstitution studies that show the LRPPRC:SLIRP complex to be essential for mtPAP to produce full length polyadenylation extensions [122]. Another important factor in the regulation of polyadenylation and mitochondrial RNA stability is the polyribonucleotide phosphorylase, PNPase. Together with the RNA helicase SUPV3L1, these molecules form the mitochondrial degradosome in the matrix [126]. This complex is essential for the removal of the many antisense RNA molecules that are generated during mtDNA transcription, but its exact role in turnover of mt-mRNAs is unclear. Originally, on the basis of many mitochondrially derived cDNA sequences deposited in the EST database, PNPase was postulated to play a key role in mtRNA turnover as so many cDNA sequences contained poly(A) sequences at the 3’ termini of transcripts from throughout the mitochondrial genome [127]. PNPase is capable of both phosphorolysis and polymerization of poly(A) sequences. Finding evidence of so much 3’ polyadenylation mimicked the situation in bacteria where RNA degradation is facilitated by PNPase synthesizing short oligo(A) sequences at 3’ termini of RNA. It was not clear, however, whether this merely represented aberrant processing, particularly as it has become clear that the default for free 3’ RNA termini without structure in mitochondria is to be polyadenylated [86–88]. Depletion of PNPase revealed a complicated dataset, with polyadenylation of some species being lost, some were unaffected and others were actually increased in length [128]. This picture was further muddled by the localization of the majority of the PNPase in the intermembrane space [129], away from where mitochondrial RNA is metabolized, although a subset of the enzyme in association with SUPV3L1 is clearly present in the matrix [126]. Depletion of mtPAP either by genetic manipulation or within cell lines of patients with deficient enzyme showed loss of the longer polyadenylated species but retained oligo(A) termini. Frustratingly, there was no clear picture, with depletion of poly(A) leading some species to be degraded rapidly, some were more stabilized and some were unaffected [122].

Mitochondrial PAP has been shown to play another key role in mitochondrial gene expression. In human mtDNA, genes encoding two mt-tRNAs (mt-tRNA^Cys^ and – tRNA^Tyr^) overlap by a single base. On separation following transcription, mt-tRNA^Tyr^ lacks the terminal discriminator base at the 3’ end. Prior to addition of the three bases by the CCA-nucleotidyltransferase, it is therefore essential that a terminal adenyl base is added, a function that is carried out by mtPAP. This job is further complicated by the inability of mtPAP to add just a single adenyl residue. Trimming of the additional residues is believed to be completed by ELAC ribonuclease Z or by the previously discussed enzyme, phosphodiesterase 12 (PDE12).

As noted above, polyadenylation of mammalian mitochondria mRNAs has differing effects depending on the transcript, but in plants polyadenylation of mt-mRNA is a signal for degradation, more closely resembling the situation in chloroplasts and some bacteria [130–132]. In trypanosomes, following trimming of mature g- or rRNA, 3’-terminal uridylation occurs but has little apparent effect. Adenylation, however, plays a much more complex role than just stability. It acts as a quality control step ensuring temporal separation between modification and translation by monitoring the pre- versus post-edited status of the mt-mRNAs [133]. Adenylation of a short (20–30 nt) tail to pre-edited RNA distinguishes them from fully edited and polyadenylated (100–300 nt) mt-mRNAs, thereby dictating the stability/translatability of the transcript (reviewed in [133]). In yeast, there is no confusion of function as they lack any adenylation of the mt-mRNAs.

## Location of events

Where it is possible, we have tried to generalize what is known about each aspect that is necessary to produce a functional RNA in various species. Where in the mitochondrial network do all these post-transcriptional events take place? In mammalian mitochondria, processing and maturation are thought to occur in membrane-less liquid condensates known as *M*itochondrial *R*NA *G*ranules (MRGs) [134,135]. In fission yeast, it has been proposed that the majority of mtDNA transcription, preparation of functional RNA and translation occurs around the nucleoid in Mitochondrial Organisation of Gene Expression (MIOREX) complexes [136], whilst in Trypanosoma, although there are complexes that act to process or mature the RNA, there is no evidence that preparation of functional RNA occurs at defined sites, similar to the case in plants.

## Conclusion

From this short review, it is clear that the molecular mechanisms that produce functional RNA for intramitochondrial translation can vary dramatically between species. Classic examples of both divergent and convergent evolution in RNA biology are displayed by these organelles. Whilst many of those functions are now being understood in detail at the molecular level, there are still many questions to answer. What regulates these processes and is regulation of functional RNA production and expression a key point in mitochondrial gene expression? There are many gaps still in our detailed knowledge of how those functional RNAs are produced in certain groups of Eukarya, or how these processes are modulated under conditions of stress. With the speed at which new technologies are becoming available, we will soon further our understanding of RNA biology in mitochondria of Eukarya.

## Data Availability

Data sharing is not applicable to this article as no new data were created or analysed in this study.

## Abbreviations

Mitochondrial: mt
ribonucleic acid: RNA
Transfer RNA: tRNA
ribosomal RNA: rRNA
guide RNA: gRNA
Deoxyribonucleic acid: DNA
oxidative phosphorylation: OXPHOS
ATP-binding cassette transporters: ABC transporters
cytochrome *b*: COB
cytochrome *c* oxidase: COX1
NADH dehydrogenase subunit 1: *nad1*
NADH dehydrogenase subunit genes: *MTND*
pentotricopeptide repeat containing protein: PPR
5-taurinomethyluridine: τm^5^U
N6 –methyladenosine: m^6^A
open reading frame: orf
Leucine Rich PentatricoPeptide Repeat Containing protein: LRPPRC
SRA Stem-Loop Interacting RNA Binding Protein: SLIRP
